# Scalable Transdiagnostic Early Assessment of Mental Health (STREAM): a study protocol

**DOI:** 10.1136/bmjopen-2024-088263

**Published:** 2024-06-13

**Authors:** Elin H Williams, Nicholas M Thompson, Gareth McCray, Maria M Crespo-Llado, Supriya Bhavnani, Diksha Gajria, Debarati Mukherjee, Teresa Del Bianco, Georgia Lockwood-Estrin, Luke Mason, Vukiwe Ngoma, Chisomo Namathanga, Richard Nkhata, Allan Bennie, Alok Ranjan, Ulemu Kawelama, Naina Midha, Anindita Singh, Innocent Mpakiza, Akshat Gautam, Sheffali Gulati, Mark H Johnson, Gillian Lancaster, Matthew K Belmonte, Emily Jones, Vikram Patel, Sharat Chandran, Emmie Mbale, Gauri Divan, Melissa Gladstone, Bhismadev Chakrabarti

**Affiliations:** 1 Centre for Autism, School of Psychology and Clinical Language Sciences, University of Reading, Reading, UK; 2 Faculty of Health, Education and Society, University of Northampton, Northampton, UK; 3 School of Medicine, Keele University, Keele, UK; 4 Department of Women and Children’s Health, Institute of Life Course and Medical Sciences, University of Liverpool, Liverpool, UK; 5 Child Development Group, Sangath, India; 6 Indian Institute of Public Health, Bengaluru, Public Health Foundation of India, Kamataka, India; 7 Centre for Brain & Cognitive Development, Birkbeck University of London, London, UK; 8 School of Social Sciences and Professions, London Metropolitan University, London, UK; 9 University of East London, London, UK; 10 Institute of Psychiatry, Psychology and Neuroscience, King's College London, London, UK; 11 Kamuzu University of Health Sciences, Blantyre, Malawi; 12 Indian Institute of Technology Bombay, Mumbai, Maharashtra, India; 13 Center of Excellence & Advanced Research for Childhood Neurodevelopmental Disorders, Child Neurology Division, Department of Paediatrics, All India Institute of Medical Sciences, New Delhi, India; 14 Department of Psychology, University of Cambridge, Cambridge, UK; 15 Primary Care & Health Sciences, Keele University, Keele, UK; 16 The Com DEALL Trust, Bengaluru, Karnataka, India; 17 Department of Psychology, Nottingham Trent University, Nottingham, UK; 18 Department of Global Health and Social Medicine, Harvard Medical School, Boston, Massachusetts, USA; 19 Department of Global Health and Population, Harvard T H Chan School of Public Health, Boston, Massachusetts, USA; 20 Department of Paediatrics, Kamuzu University of Health Sciences, Blantyre, Malawi; 21 Department of Psychology, Ashoka University, Sonipat, India; 22 India Autism Center, Kolkata, India

**Keywords:** mental health, cognition, machine learning, community child health, child & adolescent psychiatry, public health

## Abstract

**Introduction:**

Early childhood development forms the foundations for functioning later in life. Thus, accurate monitoring of developmental trajectories is critical. However, such monitoring often relies on time-intensive assessments which necessitate administration by skilled professionals. This difficulty is exacerbated in low-resource settings where such professionals are predominantly concentrated in urban and often private clinics, making them inaccessible to many. This geographic and economic inaccessibility contributes to a significant ‘detection gap’ where many children who might benefit from support remain undetected. The Scalable Transdiagnostic Early Assessment of Mental Health (STREAM) project aims to bridge this gap by developing an open-source, scalable, tablet-based platform administered by non-specialist workers to assess motor, social and cognitive developmental status. The goal is to deploy STREAM through public health initiatives, maximising opportunities for effective early interventions.

**Methods and analysis:**

The STREAM project will enrol and assess 4000 children aged 0–6 years from Malawi (n=2000) and India (n=2000). It integrates three established developmental assessment tools measuring motor, social and cognitive functioning using gamified tasks, observation checklists, parent-report and audio-video recordings. Domain scores for motor, social and cognitive functioning will be developed and assessed for their validity and reliability. These domain scores will then be used to construct age-adjusted developmental reference curves.

**Ethics and dissemination:**

Ethical approval has been obtained from local review boards at each site (India: Sangath Institutional Review Board; All India Institute of Medical Science (AIIMS) Ethics Committee; Indian Council of Medical Research—Health Ministry Screening Committee; Malawi: College of Medicine Research and Ethics Committee; Malawi Ministry of Health—Blantyre District Health Office). The study adheres to Good Clinical Practice standards and the ethical guidelines of the 6th (2008) Declaration of Helsinki. Findings from STREAM will be disseminated to participating families, healthcare professionals, policymakers, educators and researchers, at local, national and international levels through meetings, academic journals and conferences.

**STRENGTHS AND LIMITATIONS OF THIS STUDY**

- This study will develop and assess the psychometric properties of the Scalable Transdiagnostic Early Assessment of Mental Health (STREAM) digital platform, including reliability, validity and sensitivity to change.
- Data using the STREAM platform will be collected from 4000 children in India and Malawi, which vary in terms of language, culture, socioeconomic status and medical infrastructure, thereby allowing some assessment of generalisability across diverse settings.
- Convergent validity of the metrics generated by the STREAM digital platform will be assessed against a range of measures, including self-report, biological (cortisol) and neural activity (electroencephalogram).
- Community-based recruitment and the inclusion of children with known or suspected neurodevelopmental conditions will enable us to test the utility of STREAM in identifying children whose developmental status is below that expected for their age.
- The duration of the STREAM platform in its current version may pose challenges and is an area for refinement following data analysis.

## Introduction

Optimal development and good mental health in early childhood form the foundations for positive outcomes in later life, such as improved retention in education, better employment prospects and an overall higher quality of life.^1^ Accordingly, the United Nations Sustainable Development Goals Target 4.2 highlights the critical need for all children to have access to quality early life care to ensure they have the best chance of achieving their developmental potential.^2^ Accomplishing this goal necessitates the availability of appropriate tools for assessing children’s development to identify those with functional difficulties.^3 4^ Such tools can facilitate the implementation of effective interventions in early childhood, when brains are maximally plastic and responsive to changes.^5^

Many tools designed to assess difficulties in early childhood functioning face significant limitations. A majority of these tools focus on identifying or diagnosing specific neurodevelopmental conditions such as autism spectrum conditions (ASC) or intellectual disability (ID), rather than assessing neurodevelopmental status using a dimensional framework, similar to those proposed by the Research Domain Criteria^6^ and the Hierarchical Taxonomy of Psychopathology.^7^ Some tools that do measure development dimensionally, such as the Guide for Monitoring Child Development,^8^ rely only on parent-report and/or clinician-observation measures, which can be affected by recall bias, subjectivity and inter-rater variability. Critically, most tools are only applicable for a relatively narrow window within the early developmental period. For example, the recently developed Global Scales for Early Development^9 10^ and Caregiver Reported Early Development Instruments^11 12^ target only the interval from birth to 3 years of age. In contrast, the Save the Children’s International Development and Early Learning Assessment is appropriate for children aged 3–6 years.^13^ Although some tools enable assessment throughout the early developmental period (eg, Griffiths Mental Development Scales (GMDS^14^), Ages and Stages Questionnaires^15^), these are hindered by issues of scalability: they are time-intensive, proprietary and incur significant financial costs for training and implementation, requiring administration by skilled professionals. Further, the majority have been developed to high-income country norms, limiting their applicability in other contexts.^4 16 17^

These limitations of existing tools highlight a need for a tool that assesses development dimensionally, measuring functioning across multiple domains and throughout the years of early development. This need is particularly pressing in low-resource settings where access to skilled professionals may be limited. Low/middle-income countries (LMICs) face significant resource constraints^18 19^ and children in these settings are disproportionately exposed to risk factors known to impact development, including poverty, violence, inadequate hygiene and cognitive stimulation, perinatal issues, and poor nutrition.^20–23^ Such risk factors can adversely affect both physical and mental health, hinder cognitive development and contribute to poor long-term outcomes.^23 24^ These factors may partly explain why an estimated 50.2 million children in LMICs meet the criteria for some form of neurodevelopmental condition^25^ and an estimated 250 million children stand at risk of not meeting their developmental potential.^26^

The Scalable Transdiagnostic Early Assessment of Mental Health (STREAM) project aims to overcome the various limitations of existing developmental assessment tools. STREAM is a digital, tablet-based platform that assesses motor, social and cognitive functioning in children aged 0–6 years. Our objectives for the STREAM platform are that it is: (1) able to assess motor, social and cognitive abilities across the early developmental period; (2) applicable across diverse cultural settings; and (3) scalable and usable by non-specialist workers (NSWs).

We will construct normed reference curves for each developmental domain measured (motor, social, cognitive). The broader, long-term use of such reference curves is to track and identify children with atypical developmental trajectories. By enabling early identification of such children, these reference curves will facilitate timely referrals and appropriate early intervention.

### Aims

- To develop a tablet-based tool to measure motor, social and cognitive abilities of children aged 0–6 years in two low-resource settings.
- To generate normative reference curves of motor, social and cognitive abilities.
- To establish criterion validity of the domain scores (motor, social, cognitive) against an established measure of child development (GMDS).^14^
- To establish the convergent validity of the STREAM platform by assessing the relationship between domain scores and known correlates of development via self-report, biological and neural measures.
- To establish the test–retest (TR) reliability of the scores.
- To establish the responsiveness of the scores (sensitivity to change) against changes in the GMDS, in a longitudinal assessment of a subsample of children.

## Methods

### Design and study sites

The STREAM project, set in India and Malawi, is a cross-sectional study with an additional longitudinal component. Malawi is categorised as one of the world’s least developed countries, while India falls within the lower-middle income category, as defined by the Organisation for Economic Cooperation and Development.^27^ These two countries vary in terms of language, culture and medical/educational infrastructure, which enables the assessment of the STREAM platform’s potential generalisability across diverse contexts. Data collection began in March 2022 and is expected to be completed by no later than August 2024.

### Patient and public involvement

The feasibility and acceptability to stakeholders of the three established tools included within the STREAM tablet-based platform have been assessed previously.^16 28 29^ Local stakeholders within the recruitment catchment areas were consulted before commencing STREAM data collection. In India, Accredited Social Health Activists (ASHA) provided feedback on our proposed referral pathways for children and families requiring more specialist support (see the Referrals and support section). In Malawi, feedback on recruitment, acceptability, feasibility, as well as strategies for strengthening local capacity was provided by paediatricians, the Association of Early Child Development, district health officers, community health workers and local schools. Additionally, participating families provided feedback on assessment burden and feasibility of the project during piloting. These many community involvements mitigate the lack of any patient and public representative within the research team.

### Study sample

The study sample will comprise 4000 children aged between 0 and 6 years. Children will be recruited to either a *Community* (N=3700) or *Enriched* sample (N=300). The *Community* sample will consist of children recruited from Blantyre, Malawi, and New Delhi, India (specific participant recruitment protocols and selection criteria are outlined in a subsequent section). The *Enriched* sample will include children from tertiary clinical centres diagnosed with or showing characteristics indicating a high likelihood of having a neurodevelopmental condition (eg, ASC). By recruiting these children, we aim to provide a proof of principle for the reference curves generated from the STREAM platform by testing children whose developmental status is known to be below that expected for a typically developing child of their age.

### Recruitment and consent

A quota-sampling approach will be implemented to ensure adequate representation across sex and age categories (online supplemental materials 1 and 2). To monitor recruitment progress, we will conduct quarterly reviews starting from the commencement of data collection. If, during these quarterly reviews, certain age and sex categories are underrepresented in the recruited sample, targeted recruitment efforts will be undertaken.

10.1136/bmjopen-2024-088263.supp1 Supplementary data

#### *Community* sample

A database of potential participants will be established through liaison with governmental health service providers (ASHA in India and Health Surveillance Assistants in Malawi) for parents, or expecting parents, in antenatal, immunisation and weighing clinics operating within the catchment areas. Families will be approached, either at clinics, at home or by telephone, and informed of the objectives and assessment procedures of the study. Subsequent recruitment will be achieved through snowball sampling via word of mouth. Interested families will be provided with information sheets and screened for eligibility after providing informed consent. Only one child from each household will be eligible to participate.

In India, children (N=1850) aged 0–6 years will be recruited from the urban South-East District of New Delhi. In Malawi, children (N=1850) aged 0–6 years will be recruited from Limbe and Ndirande Health Centres, Blantyre District, where families typically access routine healthcare services such as vaccinations. While the majority of children attending these health centres for routine appointments in Malawi will likely be younger children (under 3 years), we will extend invitations to parents who also have other children who are older than 3 years. Children older than three may also be recruited from local primary schools and early childhood development centres.

#### *Community* sample inclusion and exclusion criteria

Children will be eligible for STREAM if:

- They are between 0 and 72 months of age (ie, 0–6 years).
- Their parent/caregiver can provide informed consent.
- They and their parent/caregiver reside within the catchment areas of the study sites.

Children will be excluded if:

- Their sibling has participated in the STREAM study.
- They have a severe vision, hearing or motor impairment, as reported by their parent/caregiver, which would limit their ability to interact with a tablet device.
- They have had an uncontrolled seizure in the last 48 hours that lasted more than 5 min.
- They are currently enrolled in another research study or trial.
- Their parent/caregiver has a severe vision or hearing impairment.
- Their parent/caregiver has a severe learning disability or a current, severe psychiatric condition.

#### *Enriched* sample

In India, children diagnosed with a neurodevelopmental condition (N=150) will be recruited through liaison with tertiary hospitals (eg, All India Institute of Medical Science; AIIMS). These children will have a pre-existing diagnosis from an experienced clinician using the Diagnostic and Statistical Manual of Mental Disorders (5th Ed; DSM-V).

Recruitment of children for the *Enriched* sample in Malawi (N=150) will target various healthcare facilities, including paediatric wards, paediatric neurology clinics, physiotherapy clinics, occupational therapy clinics, psychiatric clinics and malaria follow-up clinics, at the Queen Elizabeth Central Hospital (QECH). Additionally, children will be recruited from centres for children with special needs including the Hamilton’s Centre, Feed the Children and Jacaranda. Given resource limitations in Malawi, we anticipate that many children will not have pre-existing diagnoses of neurodevelopmental conditions. However, their attendance at these specialised clinics and centres reflects their greater likelihood of exhibiting characteristics indicative of having a neurodevelopmental condition (eg, motor or cognitive difficulties). Clinical officers will assess whether each recruited child meets the inclusion criteria for the *Enriched* sample after checking their health passport and/or medical files and observing their behaviour.

In light of variations in the typical age of onset and detection for different neurodevelopmental conditions, recruitment to the *Enriched* sample at both sites will target different phenotypes across age strata (n.b. a formal diagnosis of any neurodevelopmental condition is not necessary for inclusion in the *Enriched* sample). For instance, our recruitment strategy will target children aged 0–2 years who are either diagnosed with global developmental delay (GDD) or are exhibiting characteristics of GDD. For children aged 2–4 years, we will target those diagnosed with GDD, ASC or ID, or exhibiting characteristics of these conditions. Finally, for children aged 4–6 years, we will target those diagnosed with ASC, ID or attention deficit hyperactivity disorder (ADHD), or exhibiting characteristics of these conditions. While ADHD is typically diagnosed after 6 years of age,^30^ early signs and ‘red flags’ are often observed between 4 and 6 years.

#### *Enriched* sample inclusion and exclusion criteria

In addition to the inclusion criteria outlined above for the *Community* sample, children recruited to the *Enriched* sample must meet one of the following criteria:

- A pre-existing clinical diagnosis of a neurodevelopmental condition (eg, GDD, ID, ASD, ADHD) or
- Documented indications of developmental delays in their health passport or medical records, or such delays are observed by a clinical officer.

The criterion of being ineligible for participation if currently enrolled in another research study or trial is relaxed for the *Enriched* sample in India because of its restrictive impact on recruitment numbers as most children presenting at the tertiary clinic will likely already be accessing care.

### Primary measures assessment

**Figure 1:**
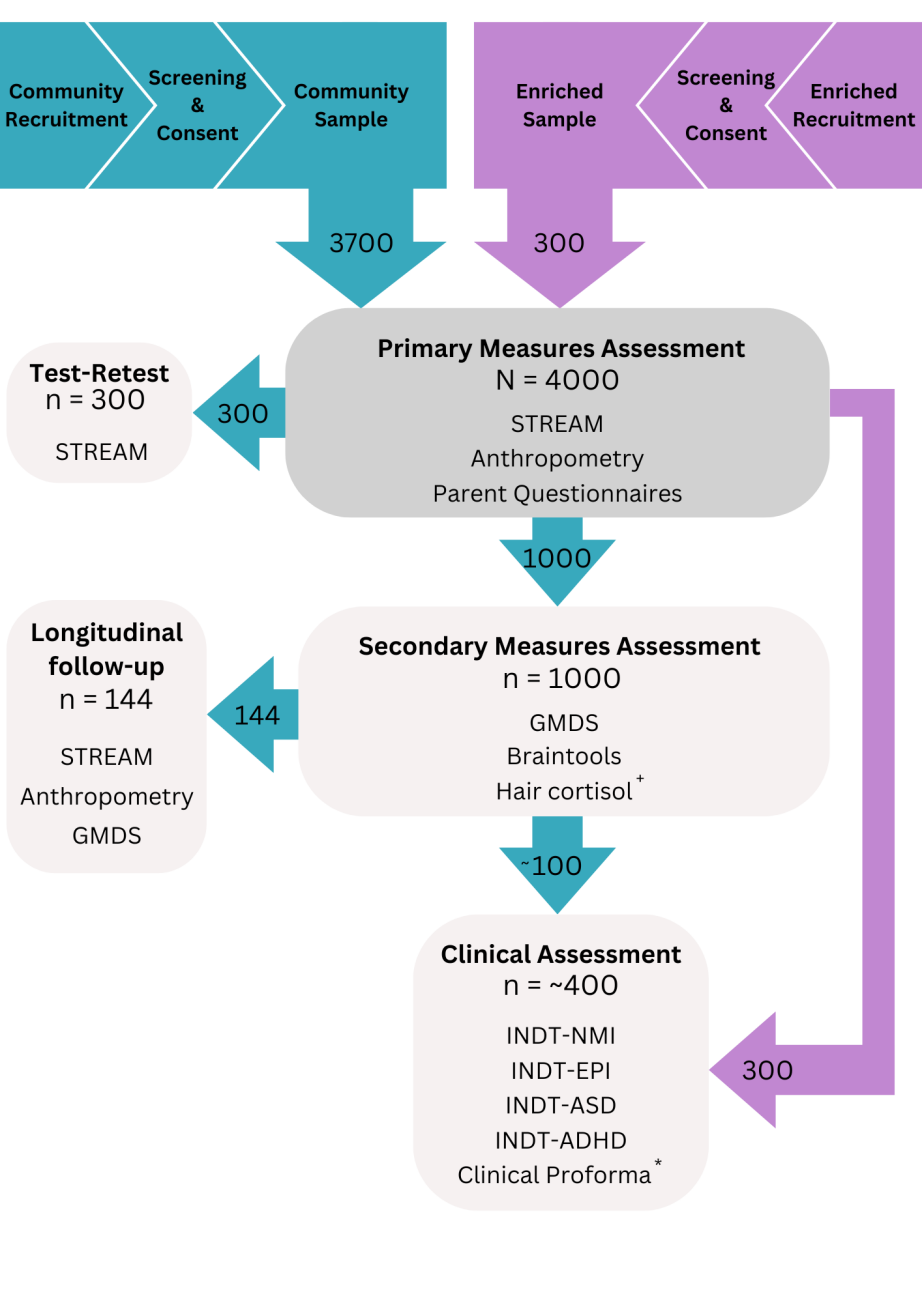
STREAM recruitment and assessment components. *As children recruited to the *Enriched* sample in Malawi are unlikely to have pre-existing diagnoses, they will additionally be administered a clinical proforma. ^+^Hair samples will be collected from a smaller subsample of n=200. GMDS, Griffiths Mental Development Scales; INDT-ADHD, INCLEN Diagnostic Tool for Attention Deficit Hyperactivity Disorder; INDT-ASD, INCLEN Diagnostic Tool for Autism Spectrum Disorder; INDT-EPI, INCLEN Diagnostic Tool for Epilepsy; INDT-NMI, INCLEN Diagnostic Tool for Neuromotor Impairment; STREAM, Scalable Transdiagnostic Early Assessment of Mental Health.

**Figure 2:**
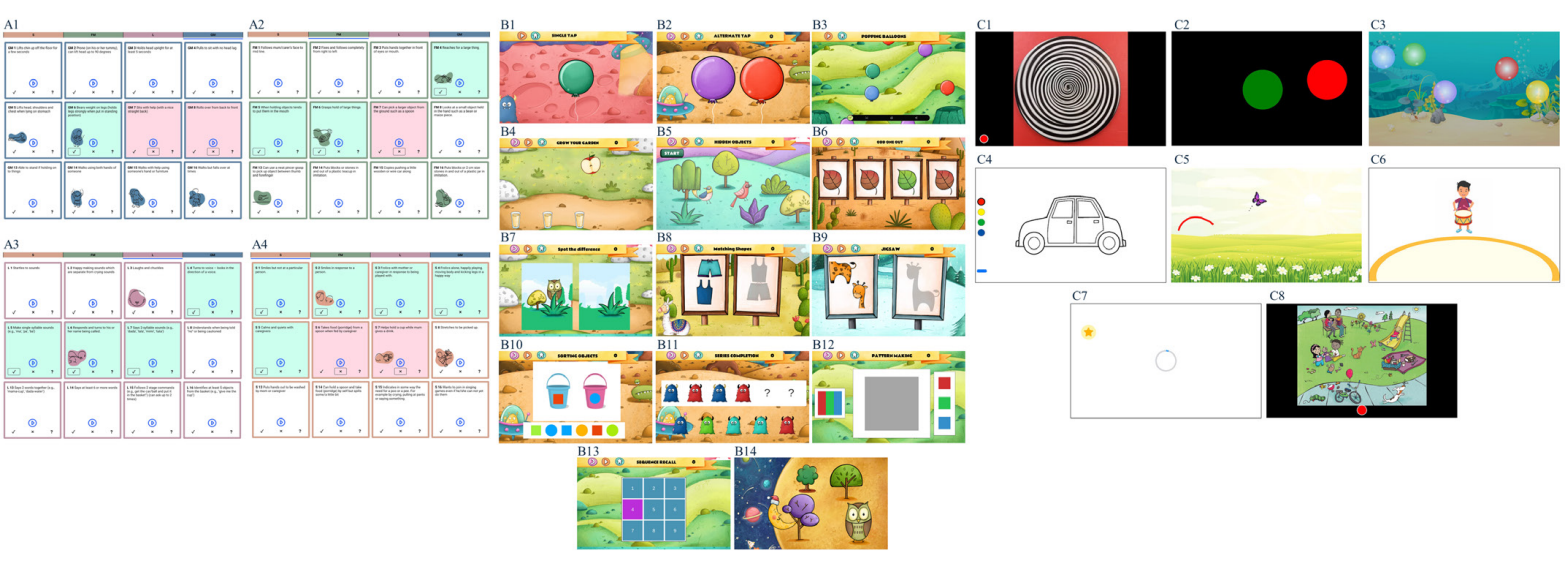
Visual depiction of each task within the Scalable Transdiagnostic Early Assessment of Mental Health platform. A: Malawi Developmental Assessment Tool (a subset of items from each grid); A1: gross motor; A2: fine motor; A3: language; A4: social. B: DEvelopmental assessment on an E-Platform; B1: single tap; B2: alternate tap; B3: popping balloons; B4: grow your garden; B5: hidden objects; B6: odd one out; B7: spot the difference; B8: matching shapes; B9: jigsaw; B10: sorting objects; B11: series completion; B12: pattern making; B13: sequence recall; B14: location recall. C: Screening Tool for Autism Risk using Technology; C1: wheel task; C2: button task; C3: bubble popping task; C4: colouring task; C5: motor following task; C6: synchrony task; C7: delayed gratification; C8: language sampling task. n.b. The images for the preferential looking task and the parent–child interaction are not included in this figure.

All (N=4000) children from the *Community* and *Enriched* samples will be administered the primary measures assessment (PMA) (figure 1) which includes (1) the STREAM platform (figure 2), (2) anthropometric measures (ie, height, weight, mid-upper arm circumference, head circumference), (3) parent/caregiver report of neurodevelopment through the Rashtriya Bal Swasthya Karyakram (RBSK)^31^ and (4) questionnaires measuring sociodemographics and exposure to risk factors relevant to neurodevelopment (details of all measures included in STREAM can be found in online supplemental material 3).

#### STREAM platform

The STREAM platform’s content and design are built on three established and complementary child development measurement tools, previously developed, field-tested and validated by the team for use in low-resource settings: the Malawi Developmental Assessment Tool (MDAT),^16^ DEvelopmental assessment on an E-Platform (DEEP)^29^ and the Screening Tool for Autism Risk using Technology (START).^28^ The MDAT is effective in identifying children aged 0–6 years with delays in social communication or motor functioning, while START and DEEP enable greater sensitivity and granularity in assessing social, motor and cognitive processes in older children (2.5–6 years), who are able to effectively interact with a tablet device independently. The integration of these tools within STREAM was piloted on N=15 children in India and N=17 in Malawi to assess the need for cultural adaptation and to inform the development of standard operating protocols (SOPs) and assessor training procedures.

#### Malawi Developmental Assessment Tool

The MDAT combines observational and performance-based assessments with parent-reported checklists, covering the following domains: gross motor, fine motor, language and social. MDAT has strong psychometric properties and serves as a reliable instrument for identifying children aged 0–6 years in low-resource settings with delayed development and/or neurodisability.^16^ Any culture-specific content within the MDAT was adapted in consultation with the tool developer, and thorough translations, back-translations and piloting were conducted at both sites prior to their inclusion within the STREAM platform.

#### DEvelopmental assessment on an E-Platform

DEEP is an innovative tablet-based tool designed to assess a range of cognitive processes such as manual speed and coordination, inhibitory control, visual perception and integration, reasoning, categorisation and memory in preschool children aged 2.5–6 years. It comprises 14 games that are integrated into an overarching storyline which aims to maximise a child’s attention and engagement with the tool. Metrics derived from children’s interaction with DEEP predict their performance on the cognitive domain of the Bayley Scales of Infant and Toddler Development (BSID-III).^32 33^

#### Screening Tool for Autism Risk using Technology

START is a mobile, modular, open-source platform originally designed for early detection of autism risk in children aged 2.5–6 years. The START tool assesses several domains associated with the autistic phenotype, including social functioning, sensory preference and fine motor skills. It uses tablet-based tasks, incorporating performance-based metrics, video-based eye-tracking and a video recording of parent–child interaction (PCI). It has shown high accuracy (>86%) in classifying children with a neurodevelopmental condition (ASC or ID) in field settings.^28^ The START stimuli have been adapted for STREAM to ensure cultural and linguistic suitability at both sites. Additionally, to facilitate the potential implementation of the STREAM platform in other contexts in the future, the stimuli for certain START tasks can be modified through the back-end content management system. Any language content has been translated, back-translated and piloted at both sites.

Each of these three tools will generate multiple output measures in STREAM (eg, a single motor function assay will generate measures of spatial and temporal error). These output measures will be combined to derive the STREAM domain scores (ie, social, cognitive, motor) (see the Statistical analysis section for specific details).

All children will be administered the MDAT observation checklists, and the START PCI component and social versus non-social preferential looking task. Children aged 2.5–6 years will additionally be administered the gamified tablet-based tasks from DEEP and START.

In order to test if a child’s performance on tablet-based assessments may be influenced by their prior exposure to smartphones or tablets, we will assess exposure through parent-report. We will allow time for children with less exposure to tablet/smartphone devices to practise using the tablet screen (eg, taps, drag and drop) before administering the STREAM tablet-based platform. During this familiarisation phase, which will last approximately 10 min, children will engage with a (non-STREAM-related) game on the device, enabling them to learn how to interact with a touch-screen device.

### Test–retest

To test the STREAM platform’s TR reliability, a subsample of n=300 children (stratified by age and sex) will be randomly selected from the *Community* sample to be re-administered the STREAM platform after an interval of 7–10 days.

### Secondary measures assessment

One thousand children from the *Community* sample, stratified by age and sex (online supplemental material 4), will be administered (1) the GMDS and (2) concurrent eye-tracking and electroencephalogram (EEG) measures (ie, Braintools). A hair sample will be collected from a smaller subsample (n=200) for the analysis of hair cortisol concentration. This secondary measures assessment (SMA) component will occur no later than 3–4 working days after the PMA.

#### Griffiths Mental Development Scales

The GMDS is widely used to measure child development across the 0–6 year age range. It provides a clinically assessed, continuous measure of strengths and needs in multiple domains (gross and fine motor coordination, language and communication, personal social-emotional function, and learning). A trained clinical psychologist or clinical officer at each site will assess each child. GMDS scores will provide dimensional measures of a child’s developmental attainment, against which the STREAM domain scores will be benchmarked as a measure of criterion validity. Although the GMDS was originally standardised in the UK, it has been widely used throughout the world.^34–36^ To ensure cultural and linguistic suitability at both sites, we have adapted some items in consultation with the tool developers (n.b. details of this adaptation work will be reported in a separate manuscript).

#### Braintools

The Braintools battery^37–39^ will use EEG to examine brain responses to social, communicational and sensory stimuli. Concurrent eye-tracking will be employed to achieve two objectives: (1) to implement gaze-contingent tasks, where trials progress when the child is attending appropriately to the stimuli presented on the computer screen and (2) to capture visual attention to social and non-social stimuli (see online supplemental material 3 for a list of Braintools tasks). The social stimuli have been adapted for cultural and linguistic suitability at both sites. The measures obtained via this component will provide a test of convergent validity for the domain scores derived from the STREAM platform. For example, we will examine the extent to which the STREAM social domain scores correlate with neural responses to social stimuli.

#### Hair cortisol

Hair samples of approximately 3 cm in length will be collected by trained assessors from n=200 children to measure hair cortisol concentration. Cortisol level in hair has been demonstrated to be a robust non-invasive indicator of stress in the preceding months and is associated with neurodevelopmental outcomes.^40^ This measure will be used to assess the convergent validity of the STREAM domain scores.

#### Clinical assessment

To understand the clinical characteristics of the *Enriched* sample at both sites, we will administer four INCLEN Diagnostic Tools (INDTs). All children aged 0–6 years in this sample will be administered the INDT for Epilepsy^41^ and INDT for Neuromotor Impairment.^42^ Children older than 12 months will be administered INDT for Autism Spectrum Disorder,^43^ and those older than 48 months will be administered INDT for Attention Deficit Hyperactivity Disorder.^44^ These tools will be administered by a trained clinical psychologist or clinical officer to either confirm the presence of a neurodevelopmental condition or characterise the nature of the functional difficulties experienced by the child. Thorough translations, back-translations and piloting of these tools has been conducted at both sites to ensure linguistic and cultural appropriateness.

As we anticipate that children recruited to the *Enriched* sample in Malawi are unlikely to have pre-existing diagnoses that could aid in characterising their functional difficulties, a clinical officer will administer a clinical proforma (online supplemental material 5) alongside the INCLEN diagnostic tools. This proforma will provide additional insights into a child’s difficulties and enable expert judgement regarding the presence/absence of a neurodevelopmental condition and the specific nature of any difficulties experienced by the child.

Furthermore, a subsample of children from the *Community* sample who have undergone both the PMA and SMA will be screened for neurodevelopmental conditions. Children who exhibit difficulties in RBSK neuromotor, motor, cognitive or social domains will be invited to participate in the clinical assessment (CA). A flag indicating higher likelihood of a neurodevelopmental condition will be assigned to children scoring 1 in any of these domains on the RBSK. Based on typical neurodevelopmental condition population prevalence rates,^45^ we expect that approximately 10% of children from the *Community* sample who have completed the SMA (n=100) will be identified as being at higher likelihood of having a neurodevelopmental condition and will be invited for this screening.

### Longitudinal follow-up

A subsample of children (n=144; stratified by age and sex) who complete the SMA will be followed up and re-administered the STREAM platform, GMDS and anthropometric measures after an 18-month interval to assess the responsiveness (sensitivity to change) of STREAM scores compared with GMDS scores.

### Assessor training

To ensure scalability, the PMA will be administered by NSWs who meet the qualifications recommended by the governments of India and Malawi for frontline health workers (ie, aged between 25 and 45 years with a minimum of senior school education). NSWs will be trained by senior project personnel in administering all components of the PMA (ie, STREAM platform, questionnaires, anthropometry). This training programme is scheduled over 8 days and involves a combination of classroom training with some explanation of child development, practical demonstrations of tool administration, and role-playing exercises. Trainees will receive feedback from trainers as well as their peers. Following this initial training, NSWs will engage in field practice for approximately 4–8 weeks to ensure they demonstrate proficiency in administering each component of the PMA and will meet regularly with trainers to address concerns or issues. Refresher training sessions will be scheduled every 3 months during the entire data collection period, although refresher training might occur more frequently during the early stages of data collection.

The SMA and CA will be conducted by clinical psychologists or clinical officers. These assessors will be trained in administering the GMDS after completion of a two-part course provided by the Association for Research in Infant and Child Development. They will be observed and evaluated by a certified GMDS trainer on at least two occasions. Braintools training will consist of multiple online tutorials complemented with a comprehensive SOP to ensure consistent execution of the protocols. Trainees will initially practise administering Braintools on adults before progressing to assessing children. All data collected by trainees will be reviewed by a senior EEG technician. Feedback on data quality will be provided, along with guidance on areas for improvement. Training for collecting hair samples for the analysis of hair cortisol concentration will consist of a 1-day online tutorial. Following this training, assessors will have access to an SOP with step-by-step instructions for sample collection. Training on the administration of the four INDTs will consist of a 2-day online tutorial provided by AIIMS. The trainee’s progress during the first few weeks of INDT administration will be supervised by a senior paediatrician. Clinical officers or psychologists will be introduced to the clinical proforma by a senior paediatrician. The senior paediatrician will provide a demonstration on how to administer and code each item of the proforma. The trainee’s progress during the first few weeks of clinical proforma administration will be supervised by the senior paediatrician.

### Data storage and quality control

Anonymised data collected offline on the STREAM platform using a tablet are encrypted and later uploaded to a secure central STREAM back-end server located in Mumbai, India. Questionnaire, anthropometry, SMA and CA data will be stored on REDCap, a web-based system for data collection,^46^ which uses a different server located in Belgium. Only approved research staff will have the requisite credentials for accessing the STREAM back-end and REDCap database.

The STREAM platform is set up as a docker container, simplifying the deployment of the back-end code on new servers for use in other projects. The platform integrates various security features to ensure compliance with the UK Data Protection Act (2018) and the EU General Data Protection Regulation. These features safeguard the security of the data whether stored on the tablet device, back-end server or during transit between the two. The STREAM platform partitions light data (eg, text, numeric) from heavy data (eg, video recordings) within the back-end server, thereby enabling more granular control over access to different types of data and optimising the efficiency of data processing and download. While light data is accessible for back-end users on logging in with the requisite credentials, heavy data on the STREAM back-end will be accessible only after the user also uploads a secret decryption key and agrees to terms and conditions for video data handling.

For monitoring field data collection, each site has a dedicated data quality officer (DQO) responsible for verifying data collected on both STREAM and REDCap platforms. The DQO will flag any instances of missing or incomplete data and report any anomalies detected in the data, thereby ensuring that potential issues with data, administration or deviations from SOPs can be addressed quickly. Bi-weekly meetings involving principal investigators, senior staff, postdocs, data staff and site personnel will monitor the ongoing progress of the project and provide a platform to address any questions or concerns raised by site teams.

### Statistical analysis

In order to optimise the information gathered from STREAM and derive three key neurodevelopmental domain scores (ie, motor, social, cognitive) and validate the platform, we will perform two largely separable analysis pathways: (1) a ‘construct-focused’ approach and (2) a machine-learning approach. These two approaches for generating the STREAM domain scores will be reviewed once both analyses have been completed. The decision as to which will constitute the primary scoring approach for STREAM will be taken at a later date and will be outlined in a subsequent paper.

#### Construct-focused score creation

In step 1 of the construct-focused method of score construction, we will collate judgements from six STREAM subject matter experts (SMEs) regarding which domain(s) each task/metric in STREAM assesses. In step 2, all output metrics from each task will be constructed using the relevant guidelines for that tool. In step 3, three factor analysis/structural equation models^47^ will be created and compared on data from n=1850 randomly subsampled children, with an n=1850 hold-out sample (n.b. The models that describe only the separate domains will effectively be separate *factor analysi*s models. Those that include a combined score will be *structural equation* models. These models will be fitted using the same set of procedures in the same statistical package. ‘Factor model’ will henceforth be used to describe both types of models). Comparing the fits of the factor models will tell us whether the domain scores are statistically distinct and whether a *total* score is justifiable. Model 1, the baseline comparator, will be unidimensional with all items loading on one factor. Model 2 will use the SME judgement data to ascribe one task to one domain (ie, no cross-loading). Model 3 will allow cross loadings in cases where the judgement data indicate that a task is measuring more than one domain. Model 4 *will not* allow cross loadings and will also include an additional second-order factor representing a ‘total score’. Model 5 *will* allow cross loadings and will also include an additional second-order factor representing a ‘total score’. Once each model is fitted, all metrics loading less than 0.34 on any factor will be removed iteratively. The inferences about the final scoring models will be based on interpretation of absolute (χ^2^, root mean square error of approximation, comparative fit index) and comparative (Akaike’s information criteria, Bayesian information criteria (BIC), adjusted BIC) model fit, using standard cut values.^48^ We will decide whether: (1) a total score is to be modelled and (2) cross-loading of tasks should be allowed. The final selected model will be fitted on the hold-out sample and the fit statistics will form evidence for structural validity. Then, the chosen model will be fitted on all data (N=3700) and the final model parameters will provide the scoring model. In line with WHO child growth standards,^49^ we will construct reference curves for age-adjusted development scores. The planned reliability and validity analyses are outlined in table 1.

**Table 1:** Overview of planned reliability and validity analysis

Evidence type	Sample size	Measure(s)	Analysis	Hypotheses
Structural validity	1850 (MDAT only) 1234 (all tools)	STREAM scores	SEMs fitted on a random n=1850, stratified by age and sex, with n=1850 hold out sample for validation	RMSEA<0.06 CFI>0.95 χ^2^
Internal consistency	4000	STREAM scores	Cronbach’s alpha (imputation of missing by design data)	Alpha>0.90* (each scale)
Test–retest reliability	300	STREAM scores	Intra-class correlation coefficient (ICC) (2, 1)	ICC (2, 1)>0.76*
Criterion validity (concurrent)	1000	GMDS scores^14^	Pearson’s correlation	R_p_>0.90* (construct focused method only)
Convergent validity (known groups) Risk of a neurodevelopmental condition	4000	RBSK scores^31^	Area under curve (AUC)	Motor>0.70* Cognitive>0.70* Social>0.70* (Total>0.70)*
Convergent validity (known groups) Stunting/malnutrition	4000	Weight for age z-score (WAZ) Height for age z-score (HAZ) Weight for height z-score (WHZ)	Area under curve (AUC)	Motor>0.70* Cognition>0.70* Social>0.70* (Total>0.70)*
Convergent validity Household SES	4000	DHS Wealth Index	Pearson’s correlation	R_p_>0.20*
Convergent validity Caregiver education	4000	Highest level of school attended	Spearman’s correlation	R_s_>0.20*
Convergent validity Caregiver depression	4000	PHQ-9^50^	Pearson’s correlation	R_p_>0.00*
Convergent validity Participation	2000	Picture My Participation (PmP)^51^	Pearson’s correlation	R_p_>0.00*
Convergent validity Exposure to violence/conflict; neglect/abuse	4000	Childhood Psychosocial Adversity Scale (CPAS)^52^	Pearson’s correlation	R_p_>0.00*
Convergent validity Home stimulation	4000	Family Care Indicators (FCI)^53^	Pearson’s correlation	R_p_>0.20*
Convergent validity Parent–child relationship	4000	Mother’s Object Relations Scale (MORS) (0–3 years old)^54^ Child Parent Relationship Scale (CPRS) (3–6 years old)	Pearson’s correlation	R_p_>0.00*
Convergent validity Social attention	1000	Various neural/eye-tracking measures of development	Pearson’s correlation	R_p_>0.00*
Convergent validity Exposure to stress	200	Hair cortisol	Pearson’s correlation	R_p_>0.00*
Responsiveness	144	STREAM GMDS, HAZ, WAZ, WHZ	Pearson’s correlation of change scores between T1 and T2 (18 m)	R_p_>0.20*

*Lower two-sided 95%CI >specified minimum.

CFI, comparative fit index; GMDS, Griffiths Mental Development Scales; MDAT, Malawi Developmental Assessment Tool; PHQ-9, Patient Health Questionnaire-9; RBSK, Rashtriya Bal Swasthya Karyakram; RMSEA, root mean square error of approximation; SES, socioeconomic status; STREAM, Scalable Transdiagnostic Early Assessment of Mental Health.

#### Machine-learning

For domain score generation, a subset of features from the STREAM platform (similar to those described in the previous paragraph on the construct-focused method) will be used, and optimisation will be performed against the target subset of the GMDS domain scores, which will serve as supervised learning labels. Our methodology will be validated by comparing the model-generated GMDS scores with the obtained GMDS scores on data in a verification set on which the machine-learning model has not been trained. Furthermore, we will scaffold the construct-focused approach of domain score generation with the machine-learning approach. Specifically, a parametric learning approach will be employed to generate scores that are correlated with seen data, thereby paving the way for future generation of construct-like scores for unseen data.

#### Subgroup analyses

All relevant statistics will be reported by site, sex and technology familiarity.

#### Sample size

Given the multiple goals of the study, multiple sample sizes have been estimated for specific purposes. A total sample size of N=4000 was found minimally sufficient to meet the various requirements of the study. This sample was broken down in multiple ways to minimise burden on individual participants and to collect the data required for analysis. For the factor model, Kline^47^ reports that a robust ratio of participants to estimated parameters is 20:1. Assuming our most complex cross-loaded model has 88 parameters (1 total score variance, 3 domain loadings, 3 correlations between domains, 27 task loadings, 27 task errors, 27 cross-loadings) this rule of thumb gives a required sample size of 1760. This is 90 less than our 1850 required to account for exclusions due to poor-quality responses. Note, all analyses address non-inferiority to specified hypotheses (see table 1) and, thus, use one-sided CIs. For internal consistency, given a sample of 4000 and assuming an alpha of 0.85, the lower bound of our one-sided 95% CI would lie at 0.84. For TR reliability, given a sample size of 300 and assuming a true reliability of 0.80, the lower bound of a one-sided 95% CI lies at 0.76. For convergent validity evidenced through area under curve (AUC), given a sample size of 4000, an expected 10% cases and assuming an AUC of 0.80, the lower bound of our one-sided 95% CI lies at 0.73. For convergent validity evidenced through Pearson’s correlations, given a sample size of 4000 and expected correlations of 0.10 and 0.30, lower one-sided 95% CIs lie at 0.07 and 0.27, respectively. The same values hold true for Spearman’s correlation, to the two decimal places reported.

## Ethics and dissemination

All STREAM components have been approved by local ethics review boards at each study site (India: Sangath Institutional Review Board; AIIMS Ethics Committee; Indian Council of Medical Research—Health Ministry Screening Committee; Malawi: College of Medicine Research and Ethics Committee; Malawi Ministry of Health—Blantyre District Health Office) and are carried out in accordance with Good Clinical Practice standards.

### Benefits for families

**Figure 3:**
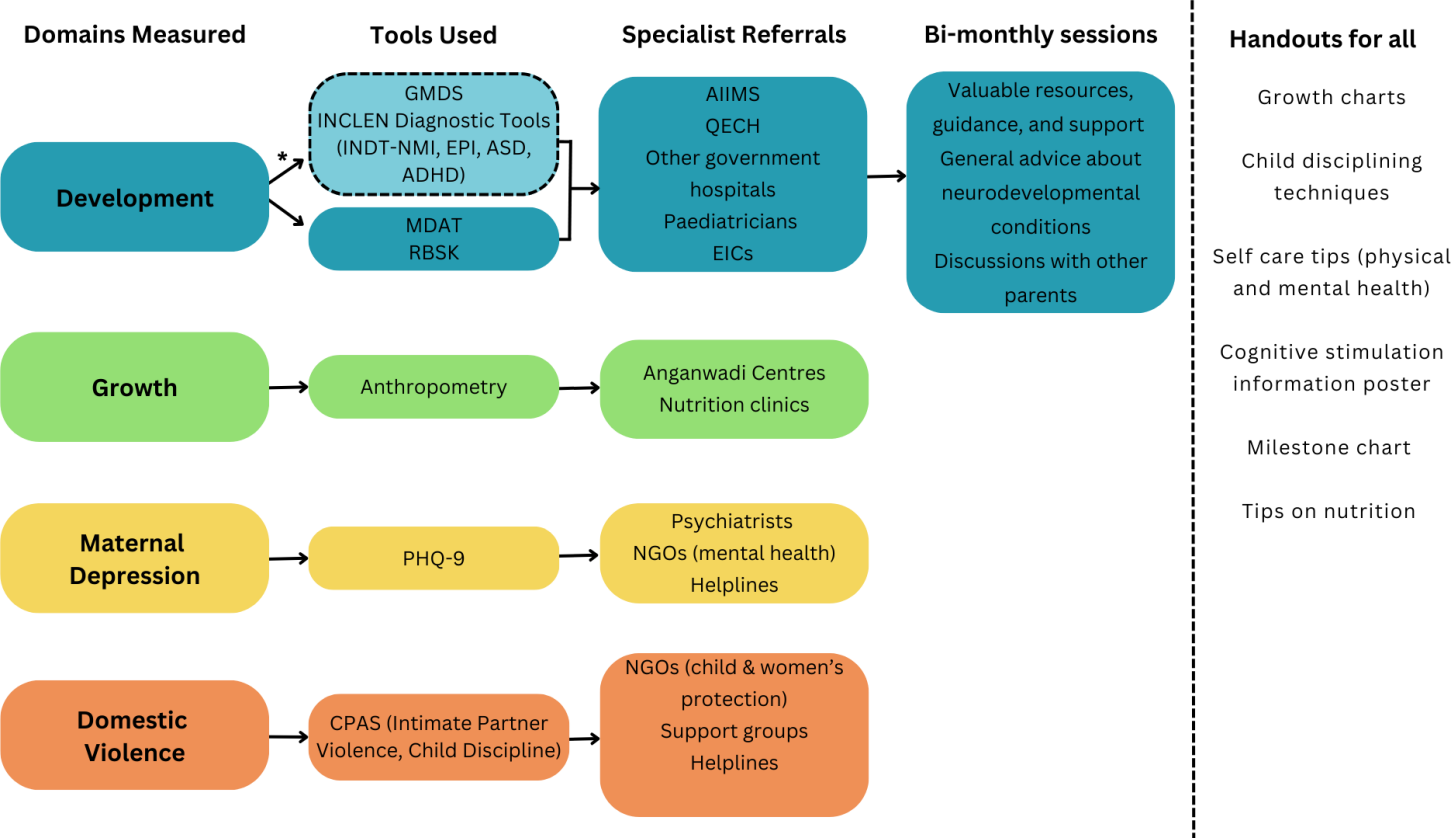
A flowchart illustrating the different referral and support pathways offered to families participating in Scalable Transdiagnostic Early Assessment of Mental Health. All families will be provided with various informative handouts. *This referral pathway is available only to children from the *Community* sample who participate in the clinical assessment. AIIMS, All India Institute of Medical Science; CPAS, Childhood Psychosocial Adversity Scale; EICs, Early Intervention Centres; GMDS, Griffiths Mental Development Scales; INDT-ADHD, INCLEN Diagnostic Tool for Attention Deficit Hyperactivity Disorder; INDT-ASD, INCLEN Diagnostic Tool for Autism Spectrum Disorder; INDT-EPI, INCLEN Diagnostic Tool for Epilepsy; INDT-NMI, INCLEN Diagnostic Tool for Neuromotor Impairment; MDAT, Malawi Developmental Assessment Tool; NGO, non-governmental organisation; PHQ-9, Patient Health Questionnaire-9; QECH, Queen Elizabeth Central Hospital; RBSK, Rashtriya Bal Swasthya Karyakram.

Each family will receive informative handouts (figure 3) covering topics such as nutrition, self-care and child discipline methods, as well as a copy of their child’s growth chart following the anthropometric assessment. Additionally, each child will be given a gift (eg, small toy, colouring pencils) and the family will receive monetary compensation for each visit (500 INR in India and 7700 MWK in Malawi) as a token of appreciation for their participation. Families will also be provided with refreshments during their visit.

### Referrals and support

The STREAM platform and various measures could potentially identify neurodevelopmental, health or social difficulties (eg, abuse or domestic violence) in children and/or their parents/caregivers. These cases may require support and referral to more specialist services via different pathways (figure 3).

Children who are identified as having height for age z-score, weight for age z-score or weight for height z-score of three SD below the WHO child growth standards^49^ will be referred to appropriate treatment services at both sites (eg, Anganwadi centres or nutrition clinics).

Children from the *Community* sample who are identified as having potential developmental delays (eg, via RBSK, MDAT, GMDS or INDTs) will receive appropriate referrals to specialist services such as tertiary hospitals (eg, AIIMS in New Delhi, QECH in Malawi), government hospitals, paediatricians and Early Intervention Centres. Parents of these children will be invited to engage in support sessions organised by the clinical psychologists on the research teams. These sessions aim to offer resources, guidance and support related to child behaviour management at home, as well as suggestions for activities to promote environmental stimulation and support cognitive development. Additionally, parents will have the opportunity to engage in interactive sessions with other parents, where they can share experiences and provide suggestions on how to manage a child’s behaviour at home.

We will refer parents/caregivers who request support or advice in relation to questionnaire items measuring exposure to violence to non-governmental organisations (NGOs) for the protection of children and women and will provide them with details of support groups and helplines to contact. We will refer mothers who are identified as having maternal depression to psychiatrists, NGOs for mental health and helplines.

### Dissemination

The STREAM project findings will be disseminated to participating families, healthcare professionals, policymakers, educators and researchers, at local, national and international levels. In Malawi, this might include the Ministry of Gender and Social Welfare, Ministry of Health and organisations working with children with special needs. In India, this might include AIIMS, Maulana Azad Medical College, India Autism Center, Ummeed Child Development Centre, Tamanna, Action for Autism, St John’s National Academy of Health Sciences ‘Unit of Hope’, ASHA and Anganwadi workers and parents of children with and without developmental disabilities. Dissemination will primarily be through meetings, academic journals and conferences.

## Supplementary Material

Reviewer comments

Author's manuscript
