# Supplementary material for: Scalable Transdiagnostic Early Assessment of Mental Health (STREAM): a study protocol
Source: BMJ Open. 2024 Jun 13;14(6):e088263. doi: 10.1136/bmjopen-2024-088263 (PMC11177698; doi:10.1136/bmjopen-2024-088263)
Supplement: Supplementary data [file bmjopen-2024-088263supp001.pdf]

Supplementary Materials

Supplementary Materials 1: Sex and age breakdown of the *Community* sample enrolled in PMA

Supplementary Materials 2: Sex and age breakdown of the *Enriched* Sample

Supplementary Materials 3. Overview of all measures included across each component of STREAM

Supplementary Materials 4: Sex and age breakdowns for the Community sample enrolled in the SMA

Supplementary Materials 5: Clinical proforma

Supplementary Materials 1: Sex and age breakdown of the *Community* sample enrolled in Primary Measures Assessment (PMA)

Community Sample

Age Group	0-3m	3-6m	6-9m	9-12m	1-1.5y	1.5-2y	2-2.5y	2.5-3y	3-3.5y	3.5-4y	4-4.5y	4.5-5y	5-5.5y	5.5-6y	**Total**
Male	77	77	77	77	154	154	154	154	154	154	154	154	155	155	**1850**
Female	77	77	77	77	154	154	154	154	154	154	154	154	155	155	**1850**
**Total**	**154**	**154**	**154**	**154**	**308**	**308**	**308**	**308**	**308**	**308**	**308**	**308**	**310**	**310**	**3700**

**To capture the rapid development occurring during the first year of life, 0 to 1 year olds will be stratified into 3-month intervals and all remaining years into 6-month intervals. The numbers specified within the table act as a general guide; it may not be feasible to recruit the exact numbers reported within each age bracket.*

Supplementary Materials 2: Sex and age breakdown of the *Enriched* Sample.

Enriched Sample

Targeted NDIs[+]	GDD	GDD, ID, ASC	ID, ASC, ADHD	
Age Group	0-2y	2-4y	4-6y	**Total**
Male	50	50	50	**150**
Female	50	50	50	**150**
**Total**	**100**	**100**	**100**	**300**

**The numbers specified within the table act as a general guide; it may not be feasible to recruit the exact numbers reported within each age bracket. [+]GDD: Global Developmental Delay; ID: Intellectual Disability; ASC: Autism Spectrum Conditions; ADHD: Attention Deficit Hyperactivity Disorder.*

Supplementary Materials 3: Overview of all measures included across each component of STREAM.

Task^	Component*	Ages	Construct Measured	Administration Mode
MDAT - Social grid	PMA, TR, & LFU	0-6	Social & communication	Caregiver report & child assessment
MDAT - Language grid	PMA, TR, & LFU	0-6	Attention & cognition	Caregiver report & child assessment
MDAT - Fine Motor grid	PMA, TR, & LFU	0-6	Motor	Caregiver report & child assessment
MDAT - Gross Motor grid	PMA, TR, & LFU	0-6	Motor	Caregiver report & child assessment
START - Preferential Looking Task	PMA, TR, & LFU	0-6	Social & communication	Child assessment
START - Parent Child Interaction	PMA, TR, & LFU	0-6	Social & communication	Child assessment
START - Button Task	PMA, TR, & LFU	2.5-6	Social & communication	Child assessment
START - Bubble Popping Task	PMA, TR, & LFU	2.5-6	Motor	Child assessment
START - Colouring Task	PMA, TR, & LFU	2.5-6	Motor	Child assessment
START - Motor Following Task	PMA, TR, & LFU	2.5-6	Motor	Child assessment
START - Synchrony Task	PMA, TR, & LFU	2.5-6	Motor	Child assessment
START - Delayed Gratification Task	PMA, TR, & LFU	2.5-6	Attention & cognition	Child assessment
START - Language Sampling Task	PMA, TR, & LFU	2.5-6	Attention & cognition	Child assessment
START - Wheel Task	PMA, TR, & LFU	2.5-6	Attention & cognition	Child assessment
DEEP - Single Tap	PMA, TR, & LFU	2.5-6	Attention & cognition	Child assessment
DEEP - Alternate Tap	PMA, TR, & LFU	2.5-6	Attention & cognition	Child assessment
DEEP - Popping Bubbles	PMA, TR, & LFU	2.5-6	Attention & cognition	Child assessment
DEEP - Grow your Garden	PMA, TR, & LFU	2.5-6	Attention & cognition	Child assessment
DEEP - Hidden Objects	PMA, TR, & LFU	2.5-6	Attention & cognition	Child assessment
DEEP - Odd one Out	PMA, TR, & LFU	2.5-6	Attention & cognition	Child assessment

**Task^**	**Component***	**Ages**	**Construct Measured**	**Administration Mode**
DEEP - Spot the Difference	PMA, TR, & LFU	2.5-6	Attention & cognition	Child assessment
DEEP - Matching Shapes	PMA, TR, & LFU	2.5-6	Attention & cognition	Child assessment
DEEP - Jigsaw	PMA, TR, & LFU	2.5-6	Attention & cognition	Child assessment
DEEP - Sorting Objects	PMA, TR, & LFU	2.5-6	Attention & cognition	Child assessment
DEEP - Series Completion	PMA, TR, & LFU	2.5-6	Attention & cognition	Child assessment
DEEP - Pattern Making	PMA, TR, & LFU	2.5-6	Attention & cognition	Child assessment
DEEP - Sequence Recall	PMA, TR, & LFU	2.5-6	Attention & cognition	Child assessment
GMDS [1]	SMA & LFU	0-6	Child development	Caregiver report & child assessment
Hair Cortisol	SMA	0-6	Child exposure to stress	N/A
Braintools - Fast ERP	SMA	0-6	Attention & cognition	Child assessment
Braintools - Social/Non-social videos	SMA	0-6	Social & communication	Child assessment
Braintools - Auditory oddball	SMA	0-6	Attention & cognition	Child assessment
Braintools - Passive scene viewing	SMA	0-6	Social & communication	Child assessment
Braintools - Frequency-tagged EEG	SMA	0-6	Attention & cognition	Child assessment
Background Information	PMA	0-6		Caregiver report
BISQ [2]	PMA	0-6	Sleep quality	Caregiver report
CPAS [3]	PMA	0-6	Security and safety	Caregiver report
Demographic & Health Scale	PMA	0-6	Child health, socio-economic status	Caregiver report
FCI [4]	PMA	0-6	Early learning opportunities	Caregiver report
INDIGO	PMA	0-6	Neurodevelopmental conditions	Caregiver report
Major life events	PMA	0-6	Major life events	Caregiver report
PHQ-9 [5]	PMA	0-6	Caregiver depression	Mother's assessment

Task^	Component*	Ages	Construct Measured	Administration Mode
RBSK [6]	PMA	0-6	Neurodevelopmental conditions	Caregiver report
MORS-SF [7]	PMA	0-3	Responsive caregiving	Caregiver report
CPRS-SF [8]	PMA	3-6	Responsive caregiving	Caregiver report
PmP [9]	PMA	3-6	Participation	Caregiver report
Anthropometry - child's mid-upper arm circumference	PMA, LFU	0-6	Child growth/nutrition	Child assessment
Anthropometry - child's head circumference	PMA, LFU	0-6	Child growth/nutrition	Child assessment
Anthropometry - child's weight	PMA, LFU	0-6	Child growth/nutrition	Child assessment
Anthropometry - child's height	PMA, LFU	0-6	Child growth/nutrition	Child assessment
Anthropometry - mother's mid-upper arm circumference	PMA, LFU	0-6	Caregiver nutrition	Mother's assessment
INCLEN - NMI [10]	CA	0-6	NMI	Caregiver report
INCLEN - EPI [11]	CA	0-6	Epilepsy	Caregiver report
INCLEN - ASD [12]	CA	1-6	ASD	Caregiver report
INCLEN - ADHD [13]	CA	4-6	ADHD	Caregiver report
Diagnosis Proforma	CA (Malawi only)	0-6	Neurodevelopmental conditions	Caregiver report & child assessment

^MDAT = Malawi Developmental Assessment Tool; START = Screening Tool for Autism Risk using Technology; DEEP = DEvelopmental assessment on an E-Platform; GMDS = Griffiths Mental Development Scales; ERP = Event related potential; EEG = electroencephalogram; BISQ = Brief Infant Sleep Questionnaire; CPAS = Child Psychosocial Adversity Scale; FCI = Family Care Indicators; PHQ-9 = Patient Health Questionnaire; RBSK = Rashtriya Bal Swasthya Karyakram; MORS-SF = Mothers' Objects Relations Scale short-form; CPRS-SF = Child-Parent Relationship Scale short-form; PmP = Picture my Participation; NMI = Neuromotor Impairment; EPI = Epilepsy; ASD = Autism Spectrum Disorder; ADHD = Attention Deficit Hyperactivity Disorder. *PMA = Primary Measures Assessment; SMA = Secondary Measures Assessment; LFU = Longitudinal follow-up; TR = Test-retest; CA = Clinical Assessment.

Supplementary Materials 4: Sex and age breakdowns for the *Community* sample enrolled in the Secondary Measures Assessment (SMA)

Community Sample

Age Group	0-3m	3-6m	6-9m	9-12m	1-1.5y	1.5-2y	2-2.5y	2.5-3y	3-3.5y	3.5-4y	4-4.5y	4.5-5y	5-5.5y	5.5-6y	**Total**
Male	21	21	21	21	41	42	41	42	41	42	41	42	42	42	**500**
Female	21	21	21	21	42	41	42	41	42	41	42	41	42	42	**500**
**Total**	**42**	**42**	**42**	**42**	**83**	**83**	**83**	**83**	**83**	**83**	**83**	**83**	**84**	**84**	**1000**

**To capture the rapid development occurring during the first year of life, 0-1 year olds will be stratified into 3-month intervals and all remaining years into 6-month intervals. The numbers specified within the table act as a general guide; it may not be feasible to recruit the exact numbers reported within each age bracket.*

Supplementary Materials 5: Clinical proforma

CLINICAL EVALUATION

**Child ID:**	
**Date of Assessment:**	
**Child's age:**	
**Exam by:**	

**Neurological Exam form**	
**1. Head abnormalities**	No abnormality ☐<br>Abnormality in shape (as seen in the pictures) ☐
**2. Eye abnormalities**	☐Yes ☐No
**3. Ear abnormalities**	☐Yes ☐No
**4. Facial dysmorphism**	☐Yes ☐No
**5. Upper or lower limb abnormalities**	☐Yes ☐No
**6. Hip dislocation**	☐Yes ☐No
**7. Scoliosis**	☐Yes ☐No
**8. Neural tube defects (spina bifida, meningocele)**	☐Yes ☐No
**9. Any features of a cleft lip or palate?**	☐Yes ☐No
**10. Any other syndromic features**	☐Yes ☐No<br>If yes, please describe _________________
**11. GMFCS level**<br><br>**To show only for children above 2 years**	**GMFCS level 1** ☐<br>**GMFCS level 2** ☐<br>**GMFCS level 3** ☐<br>**GMFCS level 4** ☐<br>**GMFCS level 5** ☐

**Diagnosis Pro-forma**	
**1. Do you think this child has a disability?**	Yes
	No
**If YES to q1**	
**1.1. How serious would you say it is?**	Mild
	Moderate
	Severe
**2. Do you think this child has a disability in any of the following areas? (Answer yes to more than one if you like)**	Motor skills
	Cognition/learning
	Hearing
	Vision
	Communication
	Socio-Emotional difficulties (e.g., severe depression or anxiety)
	Severe behavioral difficulties
	Epilepsy

**3. In your opinion as a clinician, do you think the child is suffering from any of the following neurodisabilities? (Answer yes to more than one if you like)**	
**3.1. Cerebral Palsy**	Yes
	No
	Not able to assess
**3.2. Neuromuscular condition**	Yes
	No
	Not able to assess
**3.3. Arthrogryposis**	Yes
	No
	Not able to assess
**3.4. Spina bifida**	Yes
	No
	Not able to assess
**3.5. Hydrocephalus**	Yes
	No
	Not able to assess
**3.6. Hearing impairment**	Yes, Unable to specify level
	Yes, Moderate (41-70db)
	Yes, Severe (71-90dB)
	Yes, Profound (91+dB)
	Not able to assess
**3.7. Visual impairment**	Yes, Unable to specify level
	Yes, Moderate – visual acuity of <6/60 in better eye when best corrected
	Yes, Severe – visual acuity of <3/60 in better eye when best corrected
	Not able to assess
**3.8. Global Developmental Delay (>2SD in all areas of development or half developmental age expected)**	Yes
	No
	Not able to assess
**3.9. Concerns about Autistic spectrum disorder**	Yes
	No
	Not able to assess
**3.10. Congenital developmental disorder/syndrome**	Yes
	No
	Not able to assess
**3.11. Epilepsy**	Yes
	No
	Not able to assess
**3.12. ADHD**	Yes
	No
	Not able to assess
**4. In your opinion as a clinician, what's your final diagnosis for the child?**	[Free text]
**4.1. Please, add brief explanation justifying your decision**	[Free text]

References

1. Griffiths, R. (1996). The Griffiths Mental Development Scales from birth to 2 years. *Manual. The 1996 revision Huntley: Association for Research in Infant and Child Development*.
2. Sadeh, A. (2004). A brief screening questionnaire for infant sleep problems: validation and findings for an Internet sample. *Pediatrics*, *113*(6), e570-e577. DOI: 10.1542/peds.113.6.e570
3. Berens, A. E., Kumar, S., Tofail, F., Jensen, S. K., Alam, M., Haque, R., Kakon, S. H., Petri, W. A., & Nelson III, C. A. (2019). Cumulative psychosocial risk and early child development: validation and use of the Childhood Psychosocial Adversity Scale in global health research. *Pediatric research*, *86*, 766-775. DOI: 10.1038/s41390-019-0431-7
4. Hamadani, J. D., Tofail, F., Hilaly, A., Huda, S. N., Engle, P., & Grantham-McGregor, S. M. (2010). Use of family care indicators and their relationship with child development in Bangladesh. *Journal of health, population, and nutrition*, *28*(1), 23. DOI: 10.3329/jhpn.v28i1.4520
5. Kroenke, K., Spitzer, R. L., & Williams, J. B. (2001). The PHQ-9: validity of a brief depression severity measure. *Journal of general internal medicine*, *16*(9), 606-613. DOI: 10.1046/j.1525-1497.2001.016009606.x
6. Singh, A.K., Kumar, R., Mishra, C.K., Khera, A. & Srivastava, A. (2015). Moving from survival to healthy survival through child health screening and early intervention services under Rashtriya Bal Swasthya Karyakram (RBSK). *Indian Journal of Pediatrics, 82,* 1012–1018. DOI: 10.1007/s12098-015-1823-2
7. Oates, J., Gervai, J., Danis, I., Lakatos, K., & Davies, J. (2018). Validation of the mothers' object relations scales short-form (MORS-SF). *Journal of Prenatal and Perinatal Psychology and Health*, *33*(1), 38-50
8. Pianta, R. C. (1998). *Child-Parent Relationship Scale, Short Form*. Unpublished manuscript.
9. Arvidsson, P., Dada, S., Granlund, M., Imms, C., Bornman, J., Elliott, C., & Huus, K. (2020). Content validity and usefulness of Picture My Participation for measuring participation in

children with and without intellectual disability in South Africa and Sweden. *Scandinavian Journal of Occupational Therapy*, *27*(5), 336-348. DOI: 10.1080/11038128.2019.1645878

10. Gulati, S., Aneja, S., Juneja, M., Mukherjee, S., Deshmukh, V., Silberberg, D., Bhutani, V.K., Pinto, J.M., Durkin, M., Tudu, P., Pandey, R.M., Nair, M.K.C., Arora, N.K., & INCLEN Study Group. (2014). INCLEN Diagnostic Tool for Neuromotor Impairments (INDT-NMI) for primary care physician: Development and validation. *Indian Pediatrics*, *51*(8), 613-619. DOI: 10.1007/s13312-014-0463-3
11. Konanki, R., Mishra, D., Gulati, S., Aneja, S., Deshmukh, V., Silberberg, D., Pinto, J.M., Durkin, M., Pandey, R.M., Nair, M.K.C., Arora, N.K., & INCLEN Study Group. (2014). INCLEN Diagnostic Tool for Epilepsy (INDT-EPI) for primary care physicians: Development and validation. *Indian Pediatrics*, *51*(7), 539-543. DOI: 10.1007/s13312-014-0443-7
12. Juneja, M., Mishra, D., Russell, P.S.S., Gulati, S., Deshmukh, V., Tudu, P., Sagar, R., Silberberg, D., Bhutani, V.K., Pinto, J.M., Durkin, M., Pandey, R.M., Nair, M.K.C., Arora, N.K., & INCLEN Study Group. (2014). INCLEN diagnostic tool for autism spectrum disorder (INDT-ASD): Development and validation. *Indian Pediatrics*, *51*(5), 359-365. DOI: 10.1007/s13312-014-0417-9
13. Mukherjee, S., Aneja, S., Russell, P. S., Gulati, S., Deshmukh, V., Sagar, R., Silberberg, D., Bhutani, V.K., Pinto, J.M., Durkin, M., Pandey, R.M., Nair, M.K.C., Arora, N.K. & INCLEN Study Group (2014). INCLEN diagnostic tool for attention deficit hyperactivity disorder (INDT-ADHD): Development and validation. *Indian Pediatrics*, *51*(6), 457-462. DOI: 10.1007/s13312-014-0436-6
