# Supplementary material for: Scalable Transdiagnostic Early Assessment of Mental Health (STREAM): a study protocol
Source: BMJ Open. 2024 Jun 13;14(6):e088263. doi: 10.1136/bmjopen-2024-088263 (PMC11177698; doi:10.1136/bmjopen-2024-088263)
Supplement: Reviewer comments [file bmjopen-2024-088263.reviewer_comments.pdf]

PEER REVIEW HISTORY

BMJ Open publishes all reviews undertaken for accepted manuscripts. Reviewers are asked to complete a checklist review form (**http://bmjopen.bmj.com/site/about/resources/checklist.pdf**) and are provided with free text boxes to elaborate on their assessment. These free text comments are reproduced below.

ARTICLE DETAILS

**TITLE (PROVISIONAL)**	Scalable Transdiagnostic Early Assessment of Mental health (STREAM): Study Protocol
**AUTHORS**	Williams, Elin; Thompson, Nicholas; McCray, Gareth; Crespo-Llado, Maria; Bhavnani, Supriya; Gajria, Diksha; Mukherjee, Debarati; Del Bianco, Teresa; Lockwood-Estrin, Georgia; Mason, Luke; Ngoma, Vukiwe; Namathanga, Chisomo; Nkhata, Richard; Bennie, Allan; Ranjan, Alok; Kawelama, Ulemu; Midha, Naina; Singh, Anindita; Mpakiza, Innocent; Gautam, Akshat; Gulati, Sheffali; Johnson, Mark H.; Lancaster, Gillian; Belmonte, Matthew; Jones, Emily; Patel, Vikram; Chandran, Sharat; Mbale, Emmie; Divan, Gauri; Gladstone, Melissa; Chakrabarti, Bhismadev

This article was not externally reviewed at BMJ Open. Protocols that have been independently assessed prior to submission to BMJ Open are usually fast-tracked to publication on the grounds that further substantial changes will not be possible. This independent assessment will usually be external, independent review for both a substantial grant award from a non-commercial or government funder and ethics approval.
